# Severe hypercalcemia preceding a diagnosis of *Pneumocystis jirovecii* pneumonia in a liver transplant recipient

**DOI:** 10.1186/s12879-019-4370-z

**Published:** 2019-08-22

**Authors:** Amy A. Yau, Samira S. Farouk

**Affiliations:** Division of Nephrology, Department of Medicine, Icahn School of Medicine, Mount Sinai, 1 Gustave Levy Place, New York, NY 10029 USA

**Keywords:** Hypercalcemia, Transplant, Pneumocystis, Pneumonia

## Abstract

**Background:**

Incidence of the opportunistic infection *Pneumocystis jirovecii* pneumonia (PJP) in solid organ transplant patients ranges from 5 to 15% with a mortality of up to 38%.

**Case presentation:**

We present a liver transplant recipient who developed hypoxemic respiratory failure related to PJP soon after treatment for allograft rejection. His presentation was preceded by severe hypercalcemia of 14.6 mg/dL and an ionized calcium of 1.7 mmol/L which remained elevated despite usual medical management and eventually required renal replacement therapy. As approximately 5% of PJP cases have granulomas, here we review the role of pulmonary macrophages and inflammatory cytokines in the pathophysiology of granuloma-mediated hypercalcemia. We also discuss the interpretation of our patient’s laboratory studies, response to medical therapy, and clinical risk factors which predisposed him to PJP.

**Conclusions:**

It is important for clinicians to consider PJP as an etiology of granulomatous pneumonia and non-parathyroid hormone mediated hypercalcemia in chronically immunosuppressed organ transplant recipients for timely diagnosis and management.

## Background

*Pneumocystis jirovecii* pneumonia (PJP) is an opportunistic infection with most reported cases seen in settings of immunodeficiency [1]. Incidence is noted to be 5–15% in solid organ transplant patients with a mortality of up to 38%. Pathogenesis is thought to be secondary to inhaled aerosolized fungus [2, 3]. Relative risk of infection increases up to 6 months post-transplant, and risk factors beyond the first year of transplant include increases in immunosuppression, allograft rejection, and abnormal kidney function [3, 4].

Diagnosis can be difficult given the indolent presentation. Case reports in transplant recipients describe a mild hypercalcemia preceding or occurring in conjunction with non-specific pulmonary symptoms which are later diagnosed as PJP. The hypercalcemia is thought to be mediated by pulmonary alveolar macrophages, classically seen in cases of granulomatous disease. Approximately 5% of PJP cases have granulomas seen by radiographic review or transbronchial biopsy, and even fewer patients present with pneumomediastinum [5–7].

We report a case of hypoxemic respiratory failure related to *Pneumocystis jirovecii* and pneumomediastinum in a liver transplant recipient whose diagnosis was preceded by severe hypercalcemia.

## Case presentation

We present a 70 year old male with a medical history of hepatitis B cirrhosis with a liver transplant 11 years prior to presentation, hypertension, coronary artery disease, and chronic kidney disease (CKD) stage 4/A3 who presented to our hospital for evaluation of progressive anorexia and nausea. CKD was thought to be secondary to chronic calcineurin inhibitor toxicity as well as hypertensive nephropathy. Of note, he was admitted six months prior for acute cellular allograft rejection and chronic ductopenic rejection. At that time, he received one dose of 1 g intravenous methylprednisolone with a corticosteroid taper and was started on mycophenolate mofetil (MMF) 1000 mg twice a day in addition to continuing maintenance tacrolimus.

On the day of initial presentation, vital signs were within normal limits and examination revealed a pale, thin appearing man. Cardiovascular and pulmonary examinations were normal, and the remainder of the physical exam was unremarkable. Laboratory values revealed a creatinine of 2.4 mg/dL near his prior baseline and corrected calcium of 12.0 mg/dL. The patient’s liver function tests revealed: alanine aminotransferase 48 U/L, aspartate aminotransferase 57 U/L, alkaline phosphatase 69 U/L total bilirubin 0.6 mg/dL and direct bilirubin 0.4 mg/dL respectively. The mild hypercalcemia was attributed to calcium and vitamin D supplements which were discontinued. After receiving intravenous fluids, the patient was discharged with a plan for close outpatient follow up.

He presented again seven days later with persistent failure to thrive. Physical exam was similar to prior examination. Laboratory values now revealed a corrected calcium of 14.6 mg/dL, ionized calcium of 1.7 mmol/L, and a creatinine of 2.3 mg/dL. He again received intravenous fluids. Further evaluation showed an intact parathyroid hormone (iPTH) level of less than 4 pg/mL (reference range: 10–65 pg/mL), PTH-related peptide less than 2.0 pmol/L (reference range less than 2.0 pmol/L), calcidiol (25-hydroxy vitamin D) level of 33.0 ng/mL (reference range 30–100 ng/mL), and calcitriol (1, 25-dihydroxy vitamin D) level of 72.4 pg/mL (reference range: 15–75 pg/mL). The angiotensin-converting enzyme level was 33.0 U/L (reference range: 14–82 U/L). Of note, serum protein electrophoresis showed no monoclonal protein. Serum free kappa and lambda light chains were minimally elevated at 51.0 mg/L (reference range: 3.3–19.4 mg/L) and 27.3 mg/dL (reference range: 5.6–26.3 mg/L), respectively. The day after presentation, the patient developed dyspnea and hypoxia, requiring bilevel positive airway pressure therapy. Chest x-ray showed bilateral airspace opacities, and intravenous fluids were held given concern for possible pulmonary congestion. Serum calcium remained persistently elevated despite treatment with loop diuretics, multiple doses of subcutaneous calcitonin, and 30 mg of pamidronate.

**Fig. 1:**
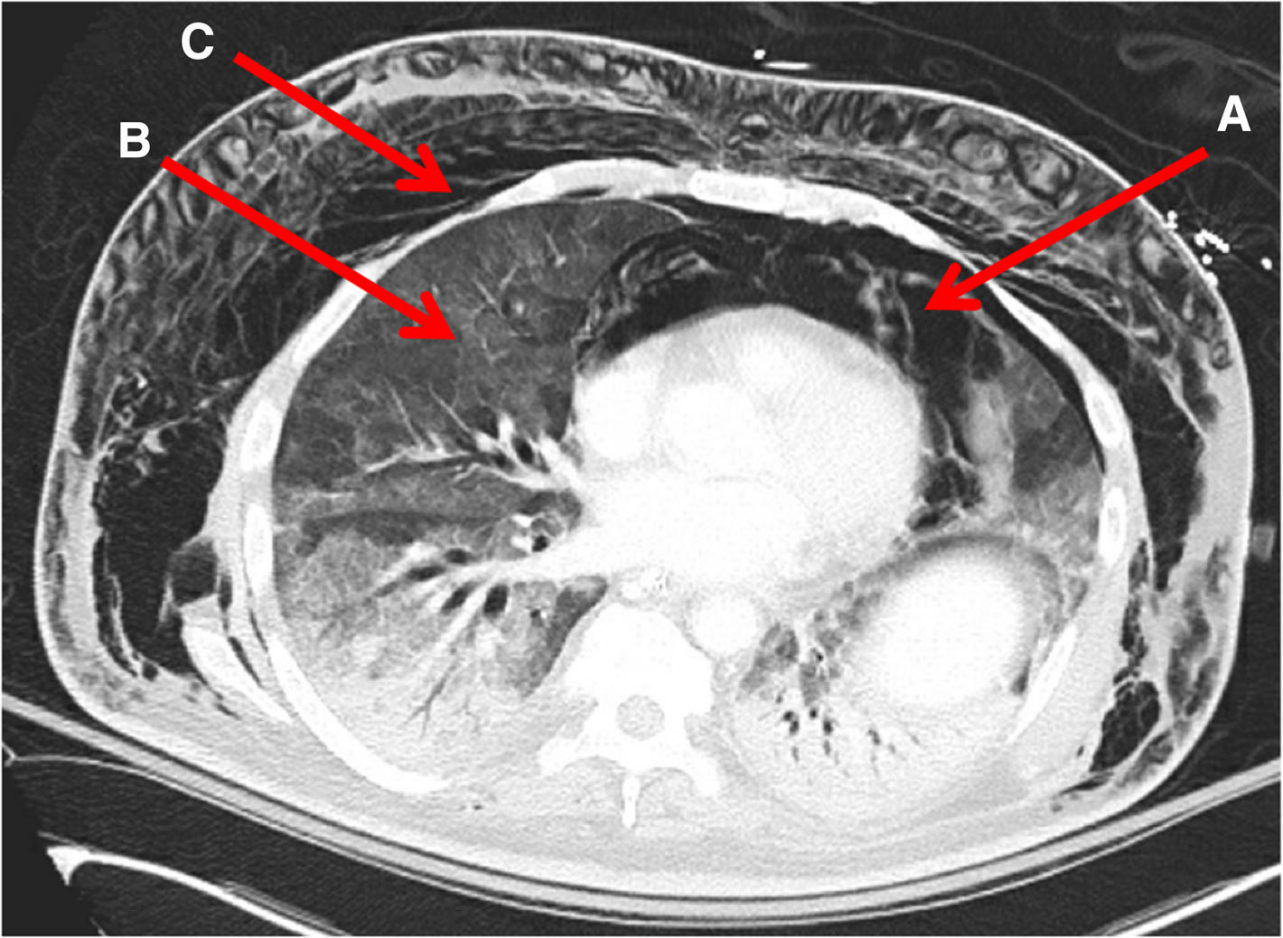
Computed Tomography of Chest, Sagittal View. **a** Severe pneumomediastinum extending from lower neck, surrounding great vessels off aortic arch and anterior portion of the heart. **b** Diffuse severe ground glass opacities in bilateral lung fields with superimposed dense consolidations with air bronchograms. **c** Extensive subcutaneous emphysema involving all fascial compartments

On day five after presentation, the patient developed worsening azotemia and now had altered mental status thought to be related to uremic encephalopathy and hypercalcemia. Dialysis was initiated for hypervolemia and uremic encephalopathy. Within a few minutes, the patient developed acute respiratory failure and hemodynamic instability requiring intubation and vasopressor support. Computed tomography of the chest revealed pneumomediastinum and ground glass opacities (Fig. 1**)**. Empiric therapy for PJP with trimethoprim-sulfamethoxazole and steroids was initiated upon transfer to the intensive care unit. Bronchoalveolar lavage confirmed a diagnosis of *Pneumocystis jirovecii*. Continuous renal replacement therapy was initiated. He remained on high dose vasopressors without clinical improvement and intensive care was withdrawn at the family’s request.

## Discussion and conclusions

A growing number of case reports and series demonstrate the relationship between non-PTH mediated hypercalcemia preceding or occurring in conjunction with a diagnosis of PJP. Most cases described in the literature occur in kidney transplant patients. To our knowledge, this is the first reported case of PJP in a non-kidney solid organ transplant with rare but classic features of pneumomediastinum as well as to hypercalcemia preceding diagnosis.

Reported risk factors for PJP include cytomegalovirus (CMV) infection, allograft rejections, glucocorticoid use, shortened or no PJP prophylaxis, age over 65 years, absolute lymphocyte count of less than 750 mm × 10^9^/L, use of MMF, induction with sirolimus, and abnormal kidney function [3, 4]. Additionally more patients with PJP had a recent history of biopsy proven rejection and treatment, most involving steroid use [4]. Our patient had similar risk factors: age over 65 years, lymphopenia, and recent steroid use and initiation of MMF for allograft rejection. Other reported cases also share a history of recent treatment for rejection or primary disease with immunosuppression increase [3, 8, 9].

Most previously reported cases had moderate non-PTH mediated hypercalcemia, [3, 6, 8, 10] with higher levels seen in patients on calcium or vitamin D supplementation, and elevated calcitriol levels [3, 9, 11, 12]. The mechanism of hypercalcemia in PJP is thought to be similar to other granulomatous diseases. Peripheral monocytes and pulmonary alveolar macrophages assist with granuloma formation and produce excess calcitriol. Moreover inflammatory cytokines, specifically interferon gamma, increase synthesis and decrease degradation of calcitriol favoring progressive hypercalcemia [8, 9, 13]. Although our patient’s calcitriol remained within the normal range, it was towards the upper limit of normal and should be considered high in the presence of renal failure and low PTH levels. As the mechanism of hypercalcemia is mediated through gut reabsorption it is not surprising our patient did not respond to bisphosphonate therapy [6, 9]. Initial hypercalcemia management should focus on administration of intravenous fluids and low calcium intake. Diuretics can be utilized in patients who develop hypervolemia. Ultimately, discovery of the etiology of hypercalcemia will guide treatment of the underlying disease process. Non-infectious, calcitriol-mediated hypercalcemia has been successfully managed with steroids and other anti-inflammatory agents, including hydroxychloroquine and infliximab.

*Pneumocystis jirovecii* pneumonia classically presents with ground glass opacities with bilateral perihilar interstitial infiltrates better described and studied in patients with poor T cell immunity secondary to the human immunodeficiency virus (HIV). Its incidence in non-HIV patients is rising and presentations in these patients tend to be more atypical making diagnosis more challenging [14]. It is hypothesized that a higher functioning immunity with preserved CD4, CD8, and adequate inflammatory cytokine production is decisive for granuloma formation [15], and reported risk factors for granuloma formation are recent corticosteroid discontinuation, HIV immune reconstitution syndrome, immunosuppression reduction, and use of aerosolized pentamidine for *Pneumocystis jirovecii* prophylaxis [11]. This may prove to be true in other non-HIV patients, as described in a patient with chronic lymphocytic leukemia whose T cell function was spared and developed granulomatous PJP [16]. The reduction in immunosuppression and improvement in T-cell mediated immunity may be drivers for granuloma formation and responsible for the subacute presentation of granulomatous PJP [11]. Understanding the immunologic factors which lead to granulomatous and non-granulomatous PJP may guide our therapy in the future regarding induction and treatment of rejection. T-cell depleting induction therapy has been associated with increased incidence of PJP [17]. Another retrospective study found that PJP incidence was increased in patients on maintenance MMF and decreased in those who received interleukin-2 receptor antagonist (IL-2RA) induction therapy, [4] yet other data suggest that MMF may actually be protective against PJP [18]. Larger studies are necessary to better identify both protective and risk factors for PJP infection. It is not surprising that in addition to more potent immunosuppression, episodes of rejection and intensification of immunosuppression are associated with PJP infection [4].

Rarely, PJP can present with barotrauma such as pneumothorax, pneumomediastinum, or subcutaneous emphysema. Though prevalence is low, mortality accompanying barotrauma is high. A review of 105 cases demonstrated 18 patients experienced barotrauma and a mortality rate of 78% [19]. The mechanism for barotrauma remains unclear, but it may be mediated by proteolytic enzymatic destruction of the lung parenchyma from alveolar macrophages, over distention of lungs via obstructive bronchiolitis, abnormal pulmonary remodeling, and direct destruction by *Pneumocystis jirovecii* [7]. Pneumomediastinum can lead to compression of the large vessels, obstructive shock, and ultimately prolonged hemodynamic instability.

Our patient’s severe hypercalcemia preceded his pulmonary symptoms by a few days, but mild hypercalcemia was present nearly two weeks prior to PJP diagnosis. It is important for clinicians to consider PJP as an etiology of granulomatous pneumonia and non-PTH mediated hypercalcemia in chronically immunosuppressed organ transplant recipients and to maintain a high index of suspicion to ensure timely diagnosis and treatment.

## Data Availability

Availability of data and material is not applicable as no datasets were generated or analysed during the current study.

## Abbreviations

CMV: cytomegalovirus
HIV: human immunodeficiency virus
IL-2RA: interleukin-2 receptor antagonist
iPTH: intact parathyroid hormone
MMF: mycophenolate mofetil
PJP: Pneymocystis jirovecci pneumonia CKD Chronic kidney disease
